# YOLO-CPCL: Compact Multi-Class Oriented Ship Detection with Adaptive Feature Fusion and Aspect-Ratio-Coupled Angle Supervision

**DOI:** 10.3390/s26154836

**Published:** 2026-07-31

**Authors:** Chenglong Ma, Shuaiqun Wang, Gele Aori, Wei Kong

**Affiliations:** 1 College of Information Engineering, Shanghai Maritime University, Shanghai 201306, China; machenglong@stu.shmtu.edu.cn (C.M.); weikong@shmtu.edu.cn (W.K.); 2 Nanjing Institute of Software Technology, Nanjing 211135, China

**Keywords:** optical remote sensing, multi-class ship detection, oriented object detection, adaptive feature fusion, angle supervision

## Abstract

Multi-class oriented ship detection in optical remote sensing images remains challenging in densely berthed and nearshore scenes. Elongated hulls, arbitrary headings, background clutter, and similar vessel appearances can weaken feature aggregation and reduce the accuracy of rotated-box localization. This study proposes YOLO-CPCL, a compact oriented detector developed from YOLOv8n-OBB. In the final YOLO-CPCL architecture, a Ship-Oriented Slender Adaptive Fusion module (SOSA-Fuse) replaces all four C2f fusion units in the Neck. It combines learned content-adaptive sampling with a C2f-style split-and-concatenation pathway to improve feature aggregation for elongated and arbitrarily oriented ships. An Aspect-Ratio-Coupled Angle Supervision Loss (ARCAS-Loss) is further introduced by applying a bounded logarithmic aspect-ratio weight to a periodic cosine angle term. This formulation strengthens angle supervision for slender targets while limiting the influence of extreme samples. On the four-class HRSC2016 task, YOLO-CPCL improves precision, recall, mAP@50, mAP@75, and mAP@50–95 by 3.54, 7.39, 5.48, 8.60, and 7.23 percentage points, respectively. The parameter count is reduced from 3.08 M to 2.92 M, corresponding to a decrease of 5.19%, while the computational cost is reduced from 8.3 to 7.6 GFLOPs, a decrease of 8.43%. Additional evaluations on the Level-2 24-class setting of ShipRSImageNet and a custom DOTA-v1.0 protocol with multi-class training and ship-class reporting show positive aggregate gains. These results demonstrate that the proposed method improves ship recall and high-IoU oriented localization while reducing the parameter count and GFLOPs.

## 1. Introduction

Optical remote sensing sensors provide wide-area observations for maritime surveillance, port traffic management, and coastal monitoring. Compared with close-range onboard sensing, satellite and aerial imagery covers large offshore and nearshore regions, but ships often occupy only a small fraction of each image and appear in cluttered surroundings. Dense berthing, sea-surface reflections, shoreline structures, and illumination changes can obscure hull boundaries or introduce responses from nearby objects [1,2]. The problem becomes more difficult when the task requires ship-type recognition and oriented localization rather than binary ship detection. Elongated hulls may appear at any heading, and visually similar vessel classes can differ only in subtle structural details [3].

The development of object detection has progressed from region-proposal methods such as R-CNN and Faster R-CNN to one-stage detectors represented by RetinaNet and YOLO [4,5,6,7]. One-stage models are attractive for maritime monitoring because they provide direct end-to-end inference with relatively modest computational cost. Their standard horizontal-box formulation, however, is poorly suited to densely arranged ships: a horizontal box often includes large areas of water or adjacent vessels. Oriented bounding boxes describe elongated targets more compactly and preserve heading information, which is useful for both instance separation and high-precision localization.

Several oriented detectors have addressed geometric representation and feature alignment. RoI Transformer learns a transformation from horizontal to rotated regions of interest [8]; S^2^A-Net aligns convolutional features with oriented anchors [9]; and R3Det progressively refines rotated predictions [10]. Ship-specific studies have also considered scale variation, weak target-background contrast, and dense target distributions. Ship-YOLO, MGA-Net, Marine-YOLO, and AR^2^Det provide representative examples for optical, SAR, and complex maritime scenes [2,3,11,12]. More recently, YOLO-LDFI integrated deformable feature operators into a compact SAR ship detector, showing the relevance of adaptive sampling to lightweight maritime perception [13]. These studies demonstrate substantial progress, but accurate multi-class detection remains difficult when elongated hulls must be localized under strict IoU criteria without increasing the model size.

Two issues are particularly relevant to the present work. First, the Neck of a compact YOLO detector aggregates features using fixed convolutional sampling. This regular grid is computationally efficient, but its local support does not adapt to variations in hull orientation or aspect ratio. In crowded ports, fixed sampling may combine responses from the ship body, water, quay structures, and neighboring vessels. Deformable convolution introduces learnable offsets that adapt the sampling pattern to image content [14,15]. The remaining challenge is to incorporate adaptive sampling into multi-scale feature fusion while retaining a compact architecture and the original C2f-style split-and-concatenation pathway.

Second, angular errors do not have the same geometric effect on all oriented boxes. For a nearly square target, a small angular deviation may have little influence on overlap. The same deviation can markedly reduce the IoU of a slender ship. Existing losses address periodicity, boundary discontinuity, and metric inconsistency through circular smooth labels, Gaussian-distance formulations, and IoU-related objectives [16,17,18]. Aspect-ratio-aware supervision has also been investigated. CBDA-Net employs an aspect-ratio-weighted angle loss [19], whereas ARS-DETR combines aspect-ratio-aware angle labels, rotated deformable attention, and dynamic angle-loss weighting for high-precision oriented detection [20]. Building on these studies, the present work employs a bounded logarithmic weighting function together with a period-consistent cosine term as auxiliary angle supervision for a compact YOLO-OBB detector.

Based on these considerations, we develop YOLO-CPCL from YOLOv8n-OBB. For brevity, the suffix CPCL denotes the combination of content-adaptive feature processing and coupled-angle learning. The detector includes two modifications that operate at different stages of the pipeline. The Ship-Oriented Slender Adaptive Fusion module (SOSA-Fuse) replaces all four C2f fusion units in the Neck and introduces learned content-adaptive sampling within a C2f-style split-and-concatenation topology. The Aspect-Ratio-Coupled Angle Supervision Loss (ARCAS-Loss) replaces the auxiliary angle-supervision term used in the adopted baseline with a bounded aspect-ratio-coupled periodic penalty. SOSA-Fuse changes feature aggregation, whereas ARCAS-Loss changes auxiliary angle supervision. Neither component adds a detection scale, and ARCAS-Loss retains the original oriented-box regression objective.

The contributions are summarized as follows:

1. A C2f-compatible SOSA-Fuse module is introduced into the multi-scale Neck. In the final YOLO-CPCL configuration, SOSA-Fuse replaces all four C2f fusion units in the Neck. The transformed branches use content-conditioned offsets for adaptive sampling, while the C2f-style split-and-concatenation pathway preserves part of the projected features for subsequent multi-scale fusion.
2. ARCAS-Loss is formulated as a bounded logarithmic aspect-ratio weight multiplied by a period-consistent cosine angle term. It provides stronger supervision for angle-sensitive slender targets without allowing extreme aspect ratios to dominate optimization.
3. YOLO-CPCL is evaluated on the four-class HRSC2016 task, the Level-2 24-class ShipRSImageNet setting, and a custom DOTA-v1.0 protocol with multi-class training and ship-class reporting. On HRSC2016, the method improves mAP@75 by 8.60 percentage points while reducing the parameter count and GFLOPs by 5.19% and 8.43%, respectively. Additional analyses characterize its class-wise behavior, statistical variation, failure modes, and practical efficiency.

The remainder of the paper is organized as follows. Section 2 reviews oriented ship detection, adaptive feature aggregation, and angle supervision. Section 3 presents the detector, datasets, and experimental protocols. Section 4 reports the experimental results. Section 5 discusses the findings and limitations, and Section 6 concludes the paper.

## 2. Related Work

### 2.1. Oriented Ship Detection in Remote Sensing Imagery

Public datasets have shaped the evaluation of oriented ship detectors. HRSC2016 provides hierarchical ship labels and rotated boxes for high-resolution optical imagery [21]. DOTA supports general oriented-object detection across a broad set of aerial scenes [22], whereas ShipRSImageNet extends optical ship detection to a fine-grained hierarchical taxonomy [23]. Oriented boxes are preferable to horizontal boxes for slender ships because they reduce background inclusion and better separate neighboring instances. Representative parameterizations and detectors include RoI Transformer, Gliding Vertex, S^2^A-Net, R3Det, and Oriented R-CNN [8,9,10,24,25]. MMRotate provides a common implementation framework for many of these models [26].

Ship-detection methods address different sensing modalities and operational conditions. E-WFF Net targets clustered optical ships and feature fusion [1]; contextual shared convolution has been used for UAV-based maritime detection [27]; MGA-Net considers multi-granularity representation in SAR scenes [12]; and Marine-YOLO addresses complex sea-surface backgrounds [2]. AR^2^Det and CBDA-Net focus on oriented localization, with the latter also introducing aspect-ratio-weighted angle supervision [3,19]. YOLO-LDFI combines a lightweight YOLO detector with deformable feature operators for SAR ship detection [13]. In contrast, the present study considers multi-class optical ship detection and couples adaptive Neck fusion with an auxiliary aspect-ratio-dependent angle term.

### 2.2. Adaptive Feature Aggregation

Feature pyramids and path aggregation are standard tools for multi-scale detection. FPN introduces top-down fusion with lateral connections [28], PANet adds a bottom-up path [29], and BiFPN uses weighted bidirectional fusion [30]. These architectures improve cross-scale information flow, but their convolutional sampling remains fixed. Deformable ConvNets learn spatial offsets from the input feature map, allowing local support to change with object geometry [14]; Deformable ConvNets V2 extends this formulation with stronger control over the sampled responses [15]. Global modeling has also been explored through DETR, Swin Transformer, and RT-DETR [31,32,33]. Multi-scale information preservation has also been studied in segmentation. SwMrNet uses multi-stage feature interaction to retain fine-grained cues in knee-tissue segmentation [34]. Although its task differs from oriented ship detection, it illustrates the broader role of information preservation in hierarchical feature aggregation. The current implementation uses the offset-only deformable convolution formulation rather than angle-guided offsets or a modulation mask. Its contribution lies in the way the operator is embedded into a C2f-style split-and-concatenation fusion unit for a compact oriented ship detector, rather than in the proposal of a new deformable-convolution operator.

### 2.3. Angle Representation and Geometry-Dependent Supervision

The optimization of rotated boxes is affected by periodicity and representation discontinuities. CSL recasts angle regression as circular classification [16]. GWD represents rotated boxes as Gaussian distributions [17], and KFIoU derives an optimizable overlap-related objective [18]. Geometry-dependent angle supervision has also been investigated. CBDA-Net applies an aspect-ratio-weighted angle loss to oriented remote sensing objects [19]. ARS-DETR further combines aspect-ratio-aware circular labels, rotated deformable attention, and dynamic angle-loss weighting, with particular emphasis on AP@75 [20]. These methods establish that aspect ratio is relevant to angular sensitivity. ARCAS-Loss differs in its use of a capped logarithmic weight, a doubled-angle cosine term, and the matching-quality weight already available in the YOLO-OBB assignment process. It replaces the auxiliary angle-supervision term used in the adopted baseline while leaving the original ProbIoU-based box-regression loss unchanged.

### 2.4. Scope of the Present Work

YOLO-CPCL addresses a specific compact-detector setting rather than proposing a general replacement for existing oriented detectors. SOSA-Fuse replaces all four C2f fusion units in the Neck and combines content-adaptive local sampling with C2f-style split-and-concatenation feature preservation. ARCAS-Loss replaces the baseline auxiliary angle term with bounded geometry-dependent supervision during training. The two components are evaluated together because one affects the features supplied to the detection head and the other affects the angular component of optimization. Claims regarding feature alignment are, therefore, limited to improved structural adaptability; the method does not impose an explicit hull-axis constraint on the learned offsets.

## 3. Materials and Methods

### 3.1. Overall Framework

YOLO-CPCL uses YOLOv8n-OBB from the Ultralytics implementation as its baseline [35]. The Backbone and oriented detection head are retained. The modifications are confined to the four C2f fusion units in the Neck and to an auxiliary training loss. This choice isolates the effects of feature aggregation and angle supervision while preserving the compact scale of the original detector.

**Figure 1:**
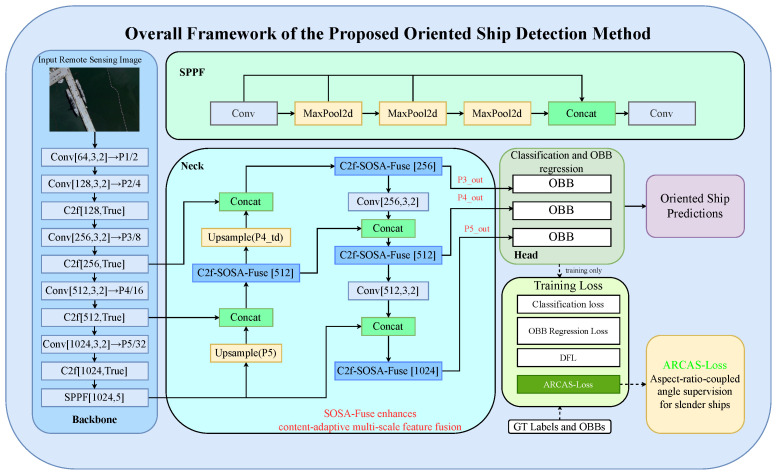
Overall framework of YOLO-CPCL. The channel values shown in the diagram denote the unscaled base configuration; the nano model uses width-scaled channels during implementation.

As shown in Figure 1, C2f-SOSA-Fuse replaces all four C2f fusion blocks distributed across the P3, P4, and P5 pathways. The module predicts offsets from the current feature map and applies deformable sampling within the transformed branches before C2f-style concatenation and fusion. ARCAS-Loss is computed for positive samples during training and replaces the auxiliary angle-supervision term used in the adopted baseline, while the original box-regression, classification, and distribution focal losses remain unchanged. The inference graph is, therefore, changed only by the Neck modules; ARCAS-Loss introduces no inference-time operation.

### 3.2. Ship-Oriented Slender Adaptive Fusion Module (SOSA-Fuse)

Ship features in remote sensing imagery are easily mixed with water texture, quay structures, and neighboring vessels, especially in dense berthing scenes. A standard convolution samples a fixed regular grid regardless of target orientation or aspect ratio. Its receptive support may, therefore, include both the hull and unrelated surroundings. This limitation concerns local feature aggregation and oriented localization; it does not, by itself, imply a direct improvement in fine-grained class separability.

SOSA-Fuse is introduced to make the local sampling support feature-dependent within all four C2f fusion units of the Neck. In C2f-SOSA-Fuse, part of the projected features is retained through the C2f-style split-and-concatenation pathway, while the transformed branch uses a learned offset field for content-adaptive sampling. The module does not use angle labels or impose an explicit hull-axis constraint. Let the input feature map be $x\in R^{c_{1}\times H\times W}$. A 1 × 1 Conv-BN-SiLU projection first produces the intermediate representation:

(1) $$x_{1}=\phi \left(x\right),$$

where ϕ(·) denotes the 1 × 1 Conv-BN-SiLU channel mapping operator. The projection sets the channel width used by the offset predictor and the deformable branch while leaving the spatial resolution unchanged.

Here, $c_{1}$, $c^{\prime }$, and $c_{2}$ denote the generic input, intermediate, and output channel widths of the SOSA-Fuse Bottleneck, respectively. In the final C2f-SOSA-Fuse implementation, each Bottleneck is instantiated with equal input and output widths *c* and an internal expansion coefficient of 1.0; therefore, $c_{1}=c^{\prime }=c_{2}=c$.

A 3 × 3 convolution then predicts the spatial offsets:

(2) $$\Delta P=\psi \left(x_{1}\right),\;\Delta P\in R^{18\times H\times W},$$

where ψ(·) is a 3 × 3 convolution and ΔP contains the horizontal and vertical offsets for the nine sampling locations. Hence, the offset tensor has 2×3×3=18 channels.

The offset field controls a 3 × 3 deformable convolution, and the output of the SOSA-Fuse Bottleneck is defined as

(3) $$y=\sigma \;\left(\mathrm{BN}\;\left(D(x_{1},\Delta P)\right)\right).$$

Here, D(·) denotes the offset-only DeformConv2d operator following Dai et al. [14], while BN(·) and σ(·) denote batch normalization and SiLU, respectively. Because the offsets are feature-dependent, the sampling support can depart from the regular convolution grid. This provides a mechanism for aggregating responses associated with ship hulls while reducing the inclusion of some irrelevant neighboring locations. However, the learned offsets are not explicitly constrained to follow the hull axis.

**Figure 2:**
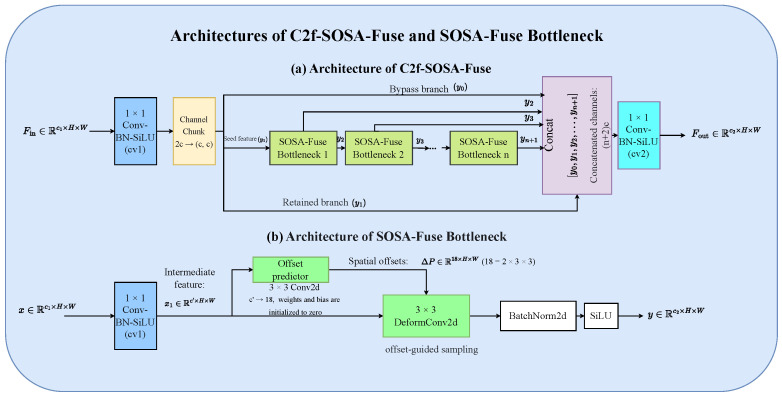
Architecture of C2f-SOSA-Fuse and SOSA-Fuse Bottleneck.

The SOSA-Fuse Bottleneck contains no element-wise input-to-output shortcut. Its output *y* is generated directly by the adaptive-sampling branch. The offset predictor is initialized to zero, so the initial sampling grid coincides with the regular convolution grid; the offsets are then learned during training. Figure 2b shows the Bottleneck structure of YOLO-CPCL.

SOSA-Fuse is then inserted into the C2f topology to form C2f-SOSA-Fuse, shown in Figure 2. Let the overall input feature be $F_{\mathrm{in}}\in R^{c_{1}\times H\times W}$, let *e* denote the channel-expansion coefficient, and let $c=\lfloor ec_{2}\rfloor$ denote the intermediate channel width of each branch after channel splitting. The splitting and recursive fusion process can be written as:

(4) $$\left\{y_{0},y_{1}\right\}=\Gamma \left(F_{\mathrm{in}}\right),\;y_{0},y_{1}\in R^{c\times H\times W};$$

(5) $$y_{k+1}=F_{\mathrm{SOSA}-B}\left(y_{k}\right),\;k=1,2,\ldots ,n;$$

(6) $$F_{\mathrm{out}}=\Theta \;\left(\mathrm{Concat}(y_{0},y_{1},y_{2},\ldots ,y_{n+1})\right),\;F_{\mathrm{out}}\in R^{c_{2}\times H\times W},$$

where Γ(·) denotes the initial splitting mapping jointly composed of the preceding 1 × 1 Conv-BN-SiLU and Channel Chunk in Figure 2a, $F_{\mathrm{SOSA}-B}\left(\cdot \right)$ denotes the SOSA-Fuse Bottleneck submodule, and Θ(·) denotes the fusion and reorganization mapping implemented by the final 1 × 1 Conv-BN-SiLU. $y_{0}$ is retained for the final concatenation, whereas $y_{1}$ is both retained and passed to the first SOSA-Fuse Bottleneck to generate $y_{2},\ldots ,y_{n+1}$. The concatenated feature, therefore, has (n+2)c channels.

C2f-SOSA-Fuse replaces, rather than supplements, all four C2f fusion units in the Neck. The intermediate width remains $c=\lfloor ec_{2}\rfloor$, and no additional feature level is introduced. In the final network, four C2f-SOSA-Fuse blocks are distributed across the P3, P4, and P5 feature-fusion pathways. The reduction in parameters and GFLOPs results from the channel configuration of the replacement blocks, whereas deformable sampling introduces additional practical runtime and memory overhead.

### 3.3. Aspect-Ratio-Coupled Angle Supervision Loss (ARCAS-Loss)

The overlap sensitivity of an oriented box depends on its shape. A small angular error has limited effect on an approximately square box but can substantially reduce the IoU of a slender box. Prior work has already established the value of aspect-ratio-aware angle supervision [19,20]. ARCAS-Loss adopts a different formulation for the present YOLO-OBB setting: a capped logarithmic aspect-ratio weight is applied to a doubled-angle cosine penalty and combined with the positive-sample matching quality. The adopted baseline already contains an auxiliary angle term based on an aspect-ratio-decayed periodic sine-squared penalty. ARCAS-Loss replaces this baseline term with the bounded logarithmic weighting and doubled-angle cosine formulation described below.

First, for the i-th positive sample, its aspect ratio is calculated according to the width and height of the ground-truth oriented bounding box:

(7) $${\mathrm{AR}}_{i}=\frac{\max(w_{i}^{\mathrm{gt}},h_{i}^{\mathrm{gt}})}{\max(\min(w_{i}^{\mathrm{gt}},h_{i}^{\mathrm{gt}}),\varepsilon )},$$

where $w_{i}^{\mathrm{gt}}$ and $h_{i}^{\mathrm{gt}}$ denote the width and height of the *i*-th ground-truth box, respectively, and ε is a very small constant used to prevent division by zero.

The geometry-dependent weight is defined as

(8) $$W_{i}^{\mathrm{ARCAS}}=\left\{\begin{array}{cc} 1+\alpha \log({\mathrm{AR}}_{i}),\; & 1+\alpha \log\left({\mathrm{AR}}_{i}\right)<W_{\max},\\ W_{\max},\; & 1+\alpha \log\left({\mathrm{AR}}_{i}\right)\geq W_{\max} \end{array}\right.,$$

where α controls the rate at which the weight grows and $W_{\max}$ caps its magnitude. The logarithm is natural, and ${\mathrm{AR}}_{i}\geq 1$ by construction. Unless otherwise stated, α=0.50 and $W_{\max}=2.0$; their sensitivity is examined in Section 4.

For completeness, the angular residual is

(9) $$\Delta \theta_{i}=\theta_{i}^{\mathrm{pred}}-\theta_{i}^{\mathrm{gt}}.$$

For the positive-sample set FG, the auxiliary loss is

(10) $$L_{\mathrm{ARCAS}}=\frac{1}{\sum_{i\in \mathrm{FG}}s_{i}}\sum_{i\in \mathrm{FG}}s_{i}\;W_{i}^{\mathrm{ARCAS}}\left[1-\cos\left(2(\theta_{i}^{\mathrm{pred}}-\theta_{i}^{\mathrm{gt}})\right)\right],$$

where $\theta_{i}^{\mathrm{pred}}$ and $\theta_{i}^{\mathrm{gt}}$ are expressed in radians, and $s_{i}$ is the positive-sample matching-quality weight produced by the assigner. The doubled angle gives a π-periodic penalty, consistent with the equivalence of the two long-axis directions. Normalization by $\sum_{i\in \mathrm{FG}}s_{i}$ keeps the scale of the term tied to the assigned positive samples.

ARCAS-Loss replaces the auxiliary angle-supervision term used in the adopted baseline but does not replace the original ProbIoU-based oriented-box regression loss. The total objective of the proposed configuration is

(11) $$L=\lambda_{\mathrm{box}}L_{\mathrm{box}}+\lambda_{\mathrm{cls}}L_{\mathrm{cls}}+\lambda_{\mathrm{dfl}}L_{\mathrm{dfl}}+\lambda_{\mathrm{ang}}L_{\mathrm{ARCAS}}.$$

Here, $L_{\mathrm{box}}$ denotes the original ProbIoU-based oriented-box regression loss, while $L_{\mathrm{cls}}$ and $L_{\mathrm{dfl}}$ denote the classification loss and Distribution Focal Loss, respectively. The corresponding coefficients are $\lambda_{\mathrm{box}}$, $\lambda_{\mathrm{cls}}$, $\lambda_{\mathrm{dfl}}$, and $\lambda_{\mathrm{ang}}$. We set $\lambda_{\mathrm{ang}}=1.0$ in the main experiments. Relative to the adopted baseline, only the formulation of the auxiliary angle-supervision term is changed. ARCAS-Loss is intended to improve angle-sensitive localization; it is not a class-balancing loss, and no claim is made that it directly resolves fine-grained category confusion.

**Figure 3:**
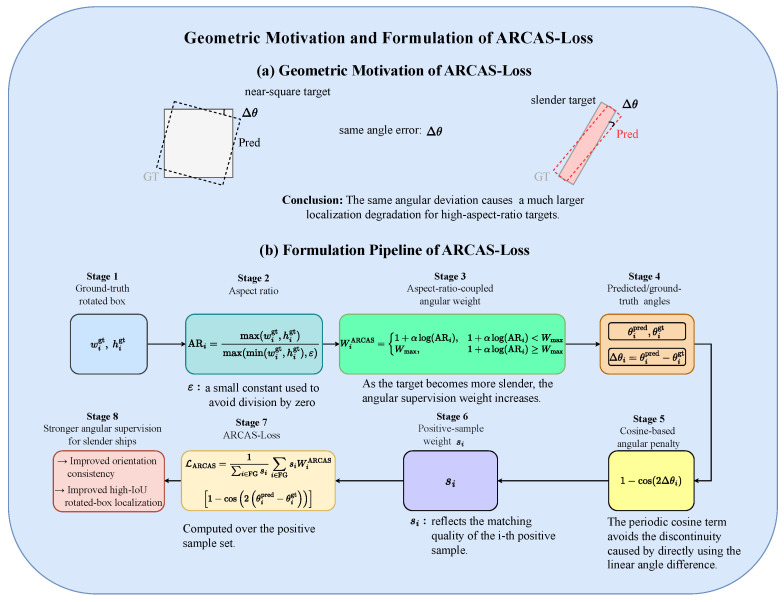
Geometric motivation and formulation of ARCAS-Loss.

Figure 3 illustrates the geometric motivation and formulation pipeline of ARCAS-Loss.

### 3.4. Relationship Between the Two Components

SOSA-Fuse and ARCAS-Loss address different parts of the detector. SOSA-Fuse changes the features propagated through the Neck by replacing fixed local sampling with learned offsets inside the transformed branches of C2f-style adaptive-fusion blocks. ARCAS-Loss operates only during training and changes the relative contribution of angular errors according to target shape. The first component can affect classification and regression through shared features, whereas the second is designed specifically for oriented localization. Their joint use is evaluated empirically in the ablation study; complementarity is inferred from the measured results rather than assumed from the architecture.

### 3.5. Datasets, Protocols, and Implementation

HRSC2016 is used for the main evaluation [21]. Following the valid-image split used in this study, the training, validation, and test sets contain 436, 181, and 444 images. The hierarchical labels are mapped to four classes: Aircraft carrier, Warship, Merchant ship, and Submarine. The label named Warcraft in the HRSC2016 hierarchy is reported as Warship. Because this four-class mapping is not the usual single-class HRSC2016 protocol, all comparisons in the paper are retrained with the same mapping and split. Table 1 lists the resulting class distribution.

ShipRSImageNet provides an additional evaluation with a larger category taxonomy [23]. We use its Level-2 24-class setting after removing DOCK; images without a remaining valid ship instance are also removed. Models are trained and evaluated on this dataset rather than transferred directly from HRSC2016, so these experiments assess applicability under a different dataset and category system, not domain generalization without retraining.

For DOTA-v1.0 [22], annotations from all 15 object categories are retained during data preparation and model training. The original training set is divided into training and validation subsets at an 80/20 ratio using a fixed random seed, while the original validation set is used as the test set. Images are center-cropped and padded where necessary to form 1024 × 1024 samples and are then converted to the YOLO-OBB format. The network input remains 640 × 640 during both training and testing. The detector is trained and evaluated in the complete 15-class setting, but only the metrics corresponding to the ship category are extracted and reported in this study. Because the data partition, preprocessing procedure, and reporting scope differ from those of the official DOTA-v1.0 benchmark, the reported ship-class results are used only for a controlled relative comparison between YOLOv8n-OBB and YOLO-CPCL and do not constitute an official DOTA benchmark or leaderboard result.

Experiments use Python 3.8.20, PyTorch 2.0.1 with CUDA 11.8, and Ultralytics 8.4.22 on a single NVIDIA GeForce GTX 1650 GPU (NVIDIA Corporation, Santa Clara, CA, USA) with 4 GB memory. The random seed is 0. cuDNN benchmarking and TF32 are disabled, cuDNN deterministic mode is enabled, and deterministic operations are requested where supported. These settings reduce, but do not eliminate, nondeterminism. Unless otherwise stated, the primary comparison and component ablations use random seed 0. To assess the statistical stability of the principal comparison, the adopted baseline and the complete YOLO-CPCL model are independently trained using seeds 0, 1, and 2. The corresponding results are reported as mean ± sample standard deviation in Section 4.7.

For HRSC2016, all models are trained from random initialization for 300 epochs at an input size of 640 × 640 and a batch size of 4. AdamW is used with an initial learning rate of 0.001 and cosine decay. Mosaic, horizontal flipping, translation, scaling, and HSV perturbation follow the Ultralytics configuration, with Mosaic disabled for the final 10 epochs. Validation is performed after each epoch, and the checkpoint selected by the Ultralytics validation fitness metric is evaluated once on the held-out test set. ShipRSImageNet and the custom DOTA-v1.0 protocol use the same optimizer, schedule, input size, and epoch count to keep the Baseline and YOLO-CPCL comparisons controlled within each evaluation setting. For the inference-efficiency evaluation, YOLOv8n-OBB and YOLO-CPCL are tested under the same software and hardware environment using an input size of 640 × 640 and a batch size of 4. The reported total latency denotes the average processing time per image, including preprocessing, model inference, and postprocessing. Throughput is calculated as the reciprocal of the total latency and is reported in frames per second (FPS). GPU memory usage is reported as the peak allocated CUDA memory during evaluation.

Table 2 summarizes the datasets, data partitions, preprocessing protocols, and roles used in this study.

Precision, Recall, mAP@50, mAP@75, and mAP@50–95 are reported together with parameter count, GFLOPs, total inference latency, throughput, and peak GPU memory usage. The two stricter metrics are emphasized because angular errors in slender boxes may have little effect at IoU = 0.50 but a much larger effect at IoU = 0.75. Precision and Recall are

(12) $$\mathrm{Precision}=\frac{\mathrm{TP}}{\mathrm{TP}+\mathrm{FP}};$$

(13) $$\mathrm{Recall}=\frac{\mathrm{TP}}{\mathrm{TP}+\mathrm{FN}},$$

where TP, FP, and FN denote true positives, false positives, and false negatives. mAP@50–95 is averaged over IoU thresholds from 0.50 to 0.95 in increments of 0.05. Parameter count and GFLOPs characterize structural complexity, whereas latency, FPS, and peak GPU memory characterize the actual runtime behavior under the tested hardware environment. These measurements are reported separately because lower theoretical complexity does not necessarily imply lower practical latency.

## 4. Results

### 4.1. Baseline Selection on HRSC2016

We first compare four nano-scale Ultralytics OBB configurations on the HRSC2016 four-class task: YOLOv8n-OBB, YOLOv11n-OBB, YOLOv12n-OBB, and YOLO26n-OBB [35]. The dataset split, input size (640 × 640), epoch count, and evaluation code are kept fixed. This comparison is used only to select the baseline architecture for the subsequent controlled experiments.

Table 3 shows that YOLOv8n-OBB gives the highest mAP@50, mAP@75, and mAP@50–95 in this comparison. YOLOv11n-OBB uses fewer parameters and FLOPs but is lower by 1.40 points at mAP@75 and 1.47 points at mAP@50–95. YOLOv12n-OBB has the highest recall, accompanied by a marked reduction in precision. YOLO26n-OBB is the smallest configuration and records the lowest accuracy values under the present training setting.

The results do not establish a general relationship between model size and ship-detection accuracy; they only show that the smaller configurations tested here do not outperform YOLOv8n-OBB under the common protocol. YOLOv8n-OBB is, therefore, retained as the baseline.

All subsequent ablations use YOLOv8n-OBB and the same training protocol.

### 4.2. Hyperparameter Sensitivity Analysis

The sensitivity of ARCAS-Loss to α and $W_{\max}$ is examined with the Baseline + ARCAS-Loss configuration. All other settings follow the HRSC2016 experiments.

Table 4 shows that the effects of α and $W_{\max}$ are metric-dependent. When $W_{\max}=2.0$, α=0.50 achieves the highest Recall and mAP@50–95 among the evaluated α values, reaching 79.15% and 63.45%, respectively. Increasing α to 0.75 or 1.00 does not further improve either metric. When α=0.50, increasing $W_{\max}$ from 2.0 to 3.0 raises mAP@75 from 72.98% to 74.52%, but decreases Recall from 79.15% to 74.74% and mAP@50–95 from 63.45% to 63.01%. Thus, the improvement at the single IoU threshold of 0.75 is accompanied by lower target recovery and lower aggregate performance across the evaluated IoU thresholds.

For the additional configurations with α=0.25, $W_{\max}=1.0$ achieves the highest Precision and mAP@50 among all evaluated settings, reaching 81.90% and 82.18%, respectively. Because the aspect-ratio-dependent weight is clipped to 1.0 for all targets in this configuration, ARCAS-Loss reduces to the unweighted periodic cosine angle-loss variant. Compared with this variant, α=0.50 and $W_{\max}=2.0$ improve Recall, mAP@75, and mAP@50–95 by 1.36, 1.98, and 0.26 percentage points, respectively, although Precision decreases. Since ARCAS-Loss is introduced to improve angle-sensitive oriented-box localization rather than to maximize Precision or mAP@50 alone, mAP@50–95 is used as the primary selection criterion, with Recall and mAP@75 serving as complementary measures of target recovery and stricter localization. Accordingly, α=0.50 and $W_{\max}=2.0$ are retained because they provide the preferred overall balance among the evaluated configurations.

The configurations with α=0.25 and $W_{\max}=2.0$ and 3.0 yield identical reported metrics. Therefore, increasing $W_{\max}$ from 2.0 to 3.0 provides no measurable benefit under the evaluated HRSC2016 setting.

### 4.3. Comparison with a Representative Oriented-Box Loss and Angle-Supervision Variants

To compare the proposed formulation with a representative oriented-box regression loss and a simpler angle-supervision variant, four controlled settings are evaluated. The adopted baseline uses the original ProbIoU-based box-regression term, distribution focal loss, classification loss, and a baseline auxiliary angle term. In the GWD variant, only the ProbIoU-based box-regression term is replaced by Gaussian Wasserstein Distance (GWD) [17], while the baseline auxiliary angle term and all other settings remain unchanged. In the simple cosine and ARCAS-Loss variants, ProbIoU is retained, whereas the baseline auxiliary angle term is replaced by an unweighted periodic cosine term or the proposed bounded aspect-ratio-coupled periodic cosine term, respectively. The model architecture, data split, initialization, assignment strategy, and training schedule remain unchanged across the comparisons. Table 5 summarizes the four controlled loss configurations.

Compared with the adopted OBB baseline, replacing ProbIoU with GWD increases Recall from 72.95% to 75.10%, corresponding to a gain of 2.15 percentage points. However, Precision, mAP@50, mAP@75, and mAP@50–95 decrease by 2.50, 1.36, 2.95, and 2.65 percentage points, respectively. Thus, GWD improves target recovery under the present experimental setting but does not produce a corresponding improvement in strict oriented-box localization.

The simple cosine variant improves Precision, Recall, mAP@50, and mAP@50–95 over the adopted OBB baseline, although its mAP@75 decreases slightly from 71.34% to 71.00%. Compared with the simple cosine variant, ARCAS-Loss increases Recall from 77.79% to 79.15%, mAP@75 from 71.00% to 72.98%, and mAP@50–95 from 63.19% to 63.45%, while Precision decreases.

ARCAS-Loss also exceeds the GWD variant by 4.05, 3.36, 4.59, and 4.35 percentage points in Recall, mAP@50, mAP@75, and mAP@50–95, respectively, whereas GWD achieves 3.91 percentage points higher Precision. Among the four settings, the simple cosine variant obtains the highest Precision and mAP@50, while ARCAS-Loss achieves the highest Recall, mAP@75, and mAP@50–95. These results show that the effects of the compared loss formulations are metric-dependent and that aspect-ratio coupling mainly benefits target recovery and localization under stricter IoU criteria rather than uniformly improving every metric.

### 4.4. Ablation Experiments on HRSC2016

Table 6 reports four controlled settings: the baseline, SOSA-Fuse alone, ARCAS-Loss alone, and their joint use. SOSA-Fuse raises recall by 6.97 points and mAP@50–95 by 4.05 points. ARCAS-Loss raises recall by 6.20 points and mAP@50–95 by 1.70 points. The joint configuration records the highest value for every reported accuracy metric.

The two single-component settings have lower precision than the baseline despite higher recall, which indicates that the additional detections include more false positives. Precision recovers in the joint setting. The parameter count decreases from 3.08 M to 2.92 M and GFLOPs from 8.3 to 7.6 whenever SOSA-Fuse is used. This reduction results from replacing the original Neck units with the narrower C2f-SOSA-Fuse configuration rather than appending a parallel module.

The larger change in mAP@75 than in mAP@50 is consistent with the intended emphasis on oriented localization. However, the ablation does not directly measure offset direction, hull-axis coverage, or angle error. The corresponding mechanism claims are, therefore, treated as interpretations of the controlled performance changes rather than direct measurements.

### 4.5. Further Ablation of SOSA-Fuse

#### 4.5.1. Contribution of Content-Adaptive Sampling

The SOSA-Fuse Bottleneck does not employ an explicit input-to-output shortcut connection. Feature reuse is instead achieved through the surrounding C2f-style split-and-concatenation topology. Accordingly, the component analysis isolates the contribution of content-adaptive sampling. In the variant without adaptive sampling, the offset predictor and deformable convolution are replaced with a standard 3×3 convolution, while the C2f topology, channel dimensions, replacement locations, training settings, and original OBB loss remain unchanged.

As shown in Table 7, the variant without adaptive sampling achieves a Precision of 80.50%, Recall of 75.30%, mAP@50 of 79.99%, mAP@75 of 72.18%, and mAP@50–95 of 61.35%. Compared with this controlled variant, the full SOSA-Fuse configuration decreases Precision by 5.47 percentage points but improves Recall, mAP@50, mAP@75, and mAP@50–95 by 4.62, 2.58, 2.77, and 4.45 percentage points, respectively. In particular, the variant without adaptive sampling does not improve mAP@50–95 over the Baseline, whereas the full SOSA-Fuse configuration increases it from 61.75% to 65.80%. Since the two variants use the same C2f-style topology, channel dimensions, replacement locations, loss function, and training protocol, these differences provide controlled evidence for the contribution of content-adaptive sampling.

#### 4.5.2. Replacement Ratio Under a Nested Location Strategy

The Neck of the adopted YOLOv8n-OBB baseline contains four replaceable C2f fusion blocks. The evaluated configurations follow a nested replacement sequence: index 15; indices 12 and 15; indices 12, 15, and 21; and all four indices 12, 15, 18, and 21. These settings correspond to replacement ratios of 25%, 50%, 75%, and 100%, respectively. The experiment, therefore, evaluates the joint effect of progressively extending the replacement scope and does not independently isolate every possible Neck location or location combination. All variants use the original OBB training loss without ARCAS-Loss, while the dataset split, channel dimensions, training schedule, and evaluation protocol remain unchanged.

As shown in Table 8, the influence of SOSA-Fuse depends on both the replacement ratio and the selected feature-fusion locations, and the metrics do not improve monotonically as the number of replaced blocks increases. The 25% configuration provides only limited changes compared with the Baseline. Increasing the replacement ratio to 50% improves Recall, mAP@50, mAP@75, and mAP@50–95, indicating that adaptive sampling at multiple feature-fusion locations is more effective than modifying a single block.

The 75% configuration achieves the highest Precision of 83.47%, together with mAP@75 and mAP@50–95 values of 74.35% and 64.17%, respectively. However, its Recall decreases to 71.22%. In contrast, the 100% configuration achieves the highest Recall, mAP@50, mAP@75, and mAP@50–95, reaching 79.92%, 82.57%, 74.95%, and 65.80%, respectively, although its Precision is lower. These results indicate that full replacement provides the most balanced improvement in target recovery and strict localization across the multi-scale Neck. Therefore, the 100% replacement configuration is adopted in the final YOLO-CPCL architecture.

### 4.6. Model Complexity and Inference Efficiency

Table 9 compares the structural complexity and practical inference efficiency of YOLOv8n-OBB and YOLO-CPCL under identical hardware and software settings.

YOLO-CPCL reduces the parameter count from 3.08 M to 2.92 M and GFLOPs from 8.3 to 7.6. The adaptive-sampling operation, however, increases total latency from 16.53 to 20.79 ms per image, reduces throughput from 60.51 to 48.09 FPS, and increases peak allocated GPU memory from 225.29 to 257.56 MB. Thus, lower structural complexity does not translate into lower end-to-end runtime or memory use on the tested GTX 1650 platform.

### 4.7. Statistical Stability of the Principal Comparison

To assess the statistical stability of the principal comparison, the adopted baseline and the complete YOLO-CPCL model are independently trained and evaluated using the same three random seeds (0, 1, and 2). Except for the random seed, the dataset split, model configuration, optimizer, training schedule, checkpoint-selection criterion, and test protocol remain unchanged. The component ablations retain the common seed-0 setting for controlled comparison.

Table 10 shows that YOLO-CPCL outperforms the adopted baseline in all five metrics for each evaluated seed. Across the three runs, the mean improvements in Precision, Recall, mAP@50, mAP@75, and mAP@50–95 are 5.39, 6.32, 7.15, 11.24, and 7.98 percentage points, respectively. YOLO-CPCL also has lower standard deviations for Recall and all three mAP metrics; the standard deviation of mAP@75 decreases from 2.38 to 0.17 percentage points. The improvement in strict oriented-box localization is, therefore, consistently observed across the evaluated seeds.

### 4.8. Class-Wise Analysis on HRSC2016

The four-class setting also allows the aggregate gains to be inspected by category. This is important because the class frequencies and visual differences are uneven, and an increase in the overall mAP can conceal declines in individual classes.

The joint model does not improve every class in every metric. The class-wise tables are, therefore, reported alongside the aggregate results, and no claim is made that the method resolves class imbalance or fine-grained category confusion.

The class-wise results in Table 11, Table 12 and Table 13 reveal a distinct category-dependent response to the proposed modifications. The most pronounced gains are observed for Aircraft carrier and Submarine. For Aircraft carrier, YOLO-CPCL improves mAP@50, mAP@75, and mAP@50–95 by 7.46, 10.67, and 9.54 percentage points, respectively, relative to the Baseline. For Submarine, the corresponding gains reach 9.85, 15.93, and 12.26 percentage points. The larger gains under mAP@75 and mAP@50–95 than under mAP@50 are consistent with improved oriented-box localization rather than only coarse target recovery. However, these metrics do not separately quantify angle, center, or side-length errors.

Warship starts from a strong Baseline, with 93.44% mAP@50 and 92.18% mAP@75. YOLO-CPCL raises these values to 94.26% and 93.24%, while its mAP@50–95 remains slightly below that of the SOSA-Fuse-only variant. The benefit of combining SOSA-Fuse and ARCAS-Loss is, therefore, limited for this class.

Merchant ship remains the lowest-performing class. YOLO-CPCL improves mAP@50, mAP@75, and mAP@50–95 by 3.80, 6.76, and 5.08 percentage points over the Baseline, but remains slightly below the ARCAS-Loss-only variant. The module gains are, therefore, category dependent rather than strictly additive. Category imbalance, intra-class variation, and visual similarity to other ship types remain important limitations.

### 4.9. Comparison with Existing Methods

Table 14 compares YOLO-CPCL with Rotated Faster R-CNN, RoI Transformer, R3Det, S^2^A-Net, and Oriented R-CNN [5,8,9,10,25]. The MMRotate implementations are used where available [26]. Each detector is trained for the same four-class task with a 640 × 640 input, and the checkpoint selected on the validation set is evaluated on the held-out test set. The backbones and detector families differ, so this experiment provides a task-level reference rather than a component-wise control.

Under the four-class HRSC2016 protocol, YOLO-CPCL records the highest mAP@50 among the methods listed in Table 14. Its parameter count and GFLOPs are also lower than those of the compared R50-based detectors and slightly lower than the YOLOv8n-OBB baseline. Because the training implementations differ across detector families, this table is used as a task-level comparison rather than a controlled architectural ablation; the controlled evidence for the two proposed components is provided in Table 6.

### 4.10. Qualitative Results, Error Analysis, and Failure Cases

**Figure 4:**
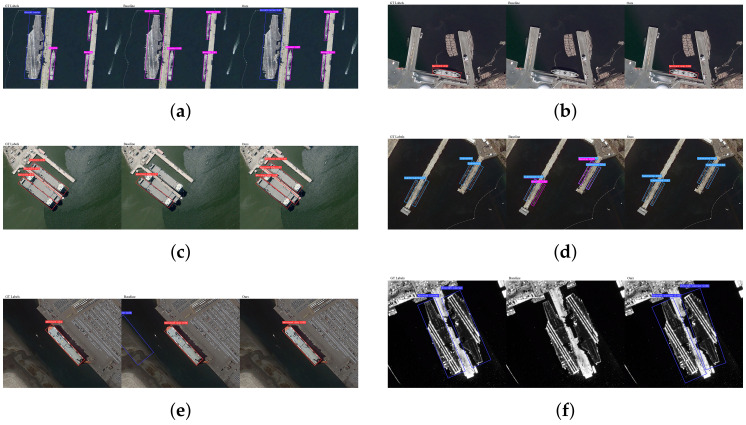
Visual comparison results of GT, Baseline, and YOLO-CPCL on the HRSC2016 test set: (**a**) category-prediction differences in a multi-class mixed scenario; (**b**) recovery of a missed single target in a low-contrast scenario; (**c**) target coverage in a multi-instance Merchant ship scenario; (**d**) semantic consistency in a challenging Submarine scenario; (**e**) suppression of false targets in a complex background; and (**f**) target detection in a large-target dual-instance scenario.

Figure 4 compares ground truth, YOLOv8n-OBB, and YOLO-CPCL on selected HRSC2016 test images. The examples include isolated ships, densely berthed targets, weak-contrast instances, and near-port backgrounds. In these cases, YOLO-CPCL recovers several targets missed by the baseline and removes some detections on non-ship structures. The figure is qualitative; the numerical changes in missed detections and background false positives are reported separately in Table 15.

Across the selected examples, the main visible differences are recovered ship instances, fewer boxes on port-side background structures, and closer agreement between predicted and annotated orientations. These observations are consistent with the higher recall and strict-IoU metrics, but they do not by themselves identify which component produces each change.

**Figure 5:**
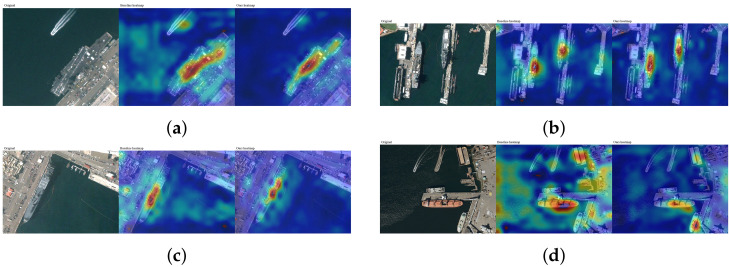
Comparison of feature response heatmaps between Baseline and YOLO-CPCL on HRSC2016 test samples: (**a**) concentration of target-body responses in a sparse scene; (**b**) preservation of target responses in a multi-instance near-port scenario; (**c**) suppression of background interference for a large-scale nearshore target; and (**d**) response distributions in a complex port background.

Figure 5 shows channel-aggregated response maps from the same intermediate feature level in the baseline and YOLO-CPCL. Each response map is resized to the input resolution and overlaid on the source image. Warmer colors indicate larger aggregated activations. Different images are used from those in Figure 4 to avoid duplicating the box-level examples.

For the displayed samples, the baseline response maps contain broader activations over water texture, shoreline structures, or port facilities. The YOLO-CPCL maps are more concentrated near several annotated ship regions. Since these maps visualize aggregated activations rather than deformable sampling offsets, they should not be interpreted as proof of hull-axis alignment. They provide qualitative support for the feature-aggregation interpretation and are considered together with the ablation and error statistics.

Table 15 groups the HRSC2016 confusion-matrix entries into four error categories. Correct detections are the diagonal entries for the four ship classes. Missed detections are ground-truth ships assigned to background; background false positives are background entries assigned to a ship class; and class confusion counts predictions assigned to the wrong ship class.

YOLO-CPCL increases correct detections from 803 to 831, while missed detections decrease from 135 to 106 and background false positives decrease from 238 to 222. Class-confusion errors change from 52 to 53. The improvement is, therefore, associated with target recovery and fewer background detections, not with a general reduction in confusion between visually similar ship classes. This distinction is consistent with the class-wise results and should be considered when interpreting the gains in mAP@75 and mAP@50–95.

**Figure 6:**
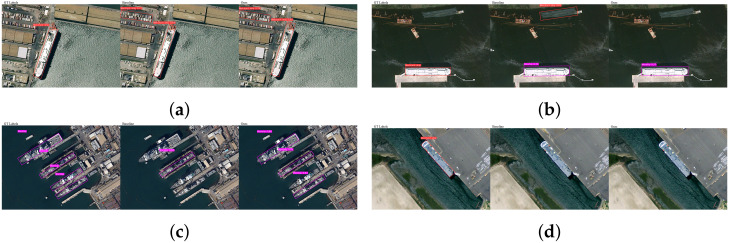
Representative failure cases of YOLO-CPCL: (**a**) a false positive on aligned land-side structures; (**b**) a merchant ship misclassified as a warship; (**c**) partial missed detections in a crowded warship scene; and (**d**) complete missed detection of a docked merchant ship.

Despite the aggregate improvements reported above, YOLO-CPCL still exhibits several representative failure modes. Figure 6 presents a background false positive, inter-class confusion, partial missed detections in a crowded warship scene, and a complete missed detection of a docked merchant ship. Each example shows the ground-truth labels, Baseline predictions, and YOLO-CPCL predictions from left to right.

In Figure 6a, YOLO-CPCL correctly detects the annotated merchant ship but also classifies a row of aligned land-side structures as another merchant ship. Their elongated arrangement, regular orientation, and repeated high-contrast appearance resemble the spatial pattern of a vessel, indicating that content-adaptive feature aggregation may still respond to ship-like non-target structures.

Figure 6b illustrates inter-class confusion. The annotated merchant ship is classified as a warship by both models. Although YOLO-CPCL suppresses the additional background response produced by the Baseline in the upper part of the scene, it does not resolve the category ambiguity of the actual vessel. This observation is consistent with the nearly unchanged class-confusion count in Table 15.

Figure 6c contains several densely berthed warships. YOLO-CPCL detects more annotated targets than the Baseline but still misses one vessel, showing that close spatial arrangement, similar appearance, and interference from neighboring ships and dock structures can hinder individual target separation. Figure 6d shows a complete missed detection of a docked merchant ship by both models. The vessel is closely connected to the quay, and its appearance partially blends with the adjacent berth and port surface. Together, these examples show that YOLO-CPCL does not completely eliminate semantic false positives, category confusion, or missed detections in crowded and structurally complex harbor environments.

### 4.11. Aspect-Ratio-Grouped Quantitative Analysis

To complement the qualitative visualizations with quantitative evidence, the ground-truth instances in the HRSC2016 test set are divided into three groups according to the aspect ratio of each oriented bounding box. For an oriented box with side lengths *w* and *h*, the aspect ratio is defined as

(14) $$\mathrm{AR}=\frac{\max(w,h)}{\min(w,h)}.$$

According to Equation (14), the ground-truth targets are divided into three groups: AR<2, 2≤AR<4, and AR≥4. Both YOLOv8n-OBB and YOLO-CPCL are evaluated at an input size of 640 × 640, using a confidence threshold of 0.001 and an oriented non-maximum-suppression IoU threshold of 0.7. Predictions are processed in descending order of confidence and matched to ground-truth boxes using class-consistent, one-to-one greedy matching based on rotated IoU. Each prediction and each ground-truth target can participate in at most one match at a given IoU threshold. Recall@50 and Recall@75 are calculated as the proportions of ground-truth targets successfully matched at rotated-IoU thresholds of 0.50 and 0.75, respectively. Matched Mean IoU is calculated only over class-consistent matches satisfying IoU≥0.50; unmatched ground-truth targets affect Recall@50 but are not included in the Matched Mean IoU calculation. The same grouping, prediction, and matching protocol is applied to both models.

As shown in Table 16, elongated ships constitute the majority of the HRSC2016 test set. Among the 990 ground-truth instances, 881 targets have AR≥4, 105 targets fall within 2≤AR<4, and only four targets have AR<2.

For the AR<2 group, both models achieve 25.00% Recall@50. YOLO-CPCL increases Recall@75 from 0.00% to 25.00% and Matched Mean IoU from 0.7051 to 0.7609. Because this group contains only four targets, these results are reported as descriptive observations and are not used to draw a general conclusion.

For targets with 2≤AR<4, YOLO-CPCL obtains a slightly lower Recall@50 than YOLOv8n-OBB, decreasing from 80.95% to 80.00%. However, Recall@75 increases from 51.43% to 61.90%, corresponding to a gain of 10.47 percentage points, while Matched Mean IoU increases from 0.7794 to 0.8099. This result indicates that the proposed method does not uniformly improve coarse target recovery in this group but improves the localization quality of successfully recovered targets under the stricter IoU criterion.

For highly elongated targets with AR≥4, YOLO-CPCL increases Recall@50 from 96.03% to 96.14%, Recall@75 from 81.61% to 86.15%, and Matched Mean IoU from 0.8358 to 0.8526. The larger improvement in Recall@75 than in Recall@50 suggests that the main benefit for slender targets lies in stricter oriented-box localization rather than merely increasing the number of coarsely detected objects. Because Matched Mean IoU excludes unmatched ground-truth targets, it should be interpreted jointly with Recall@50 and Recall@75 rather than as an independent measure of overall detection performance.

### 4.12. Ablation Experiments on ShipRSImageNet

The same ablation settings are also trained and evaluated on ShipRSImageNet. Its Level-2 taxonomy contains more categories and finer visual distinctions than the four-class HRSC2016 task. Because each configuration is retrained on ShipRSImageNet, this experiment tests whether the observed component-level trends recur under a different dataset and label system; it is not a zero-shot cross-domain evaluation.

Table 17 summarizes the ShipRSImageNet component ablation results. On this dataset, the official train/val/test split is adopted, and the Level-2 24-class setting after removing DOCK is used in terms of data organization. The experimental results show that, under the unified training and evaluation protocol on ShipRSImageNet, the proposed method improves Precision, Recall, mAP@50, mAP@75, and mAP@50–95 by 6.42, 5.26, 4.69, 4.11, and 4.06 percentage points, respectively, compared with the baseline model. This result indicates that the proposed method can still achieve positive aggregate gains in remote sensing ship scenarios with richer categories, stronger fine-grained differences, and more complex directional variations.

In the ShipRSImageNet ablation experiments, introducing ARCAS-Loss alone does not lead to simultaneous improvements across all metrics. However, the complete model achieves gains in Precision, Recall, mAP@50, mAP@75, and mAP@50–95. This indicates that, under the more complex 24-class fine-grained category system, enhancing angle supervision alone is insufficient to fully address category confusion and background interference. Content-adaptive feature aggregation in the Neck remains important for achieving consistent aggregate performance improvements.

### 4.13. Class-Wise Analysis on ShipRSImageNet

Further analysis of the category-level results on ShipRSImageNet shows that the gains are not uniform across categories. Larger improvements occur in several difficult classes, including some with low baseline AP, whereas other classes remain unchanged or decline. This pattern indicates category-dependent benefits in feature aggregation and oriented-box localization rather than a uniform improvement in fine-grained classification.

Other Merchant, Cargo, Oil Tanker, and Hovercraft do not improve in the complete model, and several additional classes fluctuate across the ablations. Table 18 and Table 19 report the category-level mAP@50 and mAP@50–95 results, respectively. Accordingly, the proposed method primarily targets oriented feature aggregation and oriented-box localization, rather than long-tail or fine-grained classification.

The mAP@50–95 results in Table 19 further reveal that the aggregate improvement is driven by several categories that are difficult for the Baseline. Commander exhibits the largest gain, increasing from 21.74% to 56.86%, followed by Patrol from 6.28% to 20.86%, Auxiliary Ships from 46.95% to 57.77%, and Tugboat from 24.38% to 32.80%. Improvements are also observed for Cruiser, Frigate, Container Ship, Sailboat, and Motorboat. The persistence of these gains under mAP@50–95 demonstrates that the improvements are not confined to detections accepted at a relaxed IoU threshold, but also extend to localization quality across stricter evaluation thresholds.

The category-level ablations also show that SOSA-Fuse and ARCAS-Loss are complementary at the aggregate level but are not strictly additive for every class. SOSA-Fuse alone achieves the highest mAP@50–95 for Cruiser, Destroyer, Frigate, Submarine, Other Merchant, RoRo, Cargo, Barge, Yacht, Fishing Vessel, Oil Tanker, and Other Ship, whereas the complete model performs best for Aircraft carrier, Patrol, Landing, Commander, Auxiliary Ships, Other Warship, Container Ship, Tugboat, Sailboat, and Motorboat. This distribution shows that the two components affect the class-wise performance pattern differently, but it does not establish a direct relationship between the gains and specific ship geometries.

Several categories remain challenging. Relative to the Baseline, YOLO-CPCL decreases mAP@50–95 from 15.10% to 14.01% for Other Merchant, from 57.23% to 53.36% for Cargo, from 46.56% to 43.43% for Oil Tanker, and from 65.31% to 64.41% for Hovercraft. Decreases are also observed for Ferry, Fishing Vessel, and Other Ship. These results expose a limitation of the current design: improving oriented feature aggregation and angle-sensitive localization does not consistently improve fine-grained discrimination among categories with overlapping visual characteristics. The aggregate gains on ShipRSImageNet, therefore, coexist with unresolved category imbalance and inter-class confusion.

### 4.14. Visual Analysis on ShipRSImageNet

**Figure 7:**
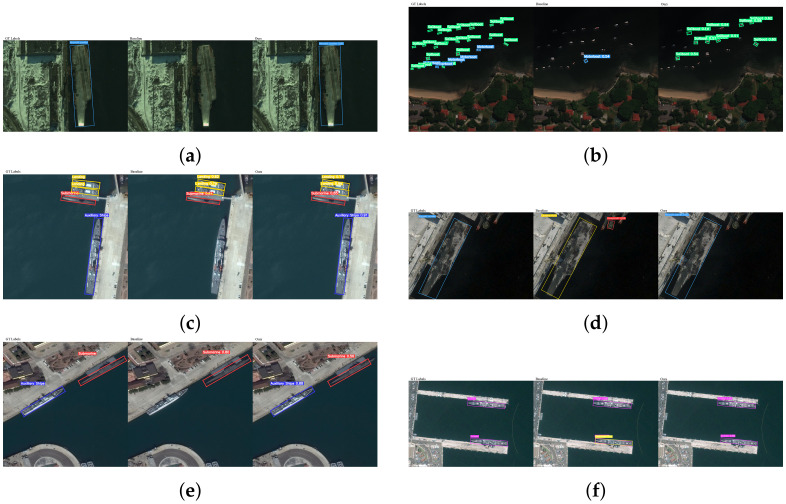
Visual comparison results of GT, Baseline, and YOLO-CPCL on the ShipRSImageNet test set: (**a**) recovery of missed detections in a single-target scenario and correct detection of a large elongated target; (**b**) improved recall in a low-illumination dense small-target scenario; (**c**) complete detection of multiple categories in a complex berth scenario; (**d**) category correction and false-target suppression in a large-target scenario; (**e**) recovery of missed detections and consistent category prediction in a multi-class scene; and (**f**) category consistency in a same-class dual-instance scenario.

Figure 7 presents representative ShipRSImageNet test images. The dataset contains a broader category hierarchy and more varied scene layouts than the four-class HRSC2016 task, allowing the detection behavior of the same architectural modifications to be examined under a different training and evaluation setting.

In these examples, YOLO-CPCL recovers several instances missed by the baseline and removes some background detections. The examples are qualitative and do not establish generalization by themselves; they are consistent with the aggregate recall and mAP results reported above.

### 4.15. Results Under the Custom DOTA-v1.0 Protocol

A final experiment evaluates the proposed modifications under a custom DOTA-v1.0 protocol. Both YOLOv8n-OBB and YOLO-CPCL are trained using annotations from all 15 DOTA-v1.0 categories, whereas only the evaluation metrics for the ship category are reported. Because the data split, preprocessing procedure, and reporting scope differ from those of the official DOTA benchmark, the results are used only for a controlled relative comparison between the two models and are not directly comparable with official DOTA leaderboard results.

The custom split and preprocessing procedures are described in Section 3.5. Both models use identical 640 × 640 inputs, training schedules, category annotations, checkpoint-selection procedures, and evaluation code. The comparison, therefore, isolates the relative effect of the proposed modifications within this custom experimental setting.

Table 20 reports the ship-class results under the custom DOTA-v1.0 protocol. For the ship category, YOLO-CPCL exceeds the Baseline by 0.40, 2.55, 1.00, 2.68, and 2.07 percentage points in Precision, Recall, mAP@50, mAP@75, and mAP@50–95, respectively. The larger gains at mAP@75 and mAP@50–95 indicate a stronger relative improvement in ship localization under stricter IoU criteria. These results describe ship-class performance within a detector trained on all 15 categories and should not be treated as results from a ship-only training setting.

## 5. Discussion

The experimental results indicate that the improvement of YOLO-CPCL mainly comes from the interaction between adaptive feature aggregation and geometry-sensitive angle supervision. In the HRSC2016 ablation study, SOSA-Fuse contributes the larger individual gain, especially in recall and mAP@50–95. This suggests that modifying the Neck feature fusion is useful for preserving elongated ship responses in cluttered port and nearshore scenes. ARCAS-Loss brings a smaller but still positive gain in the stricter localization metrics, which is consistent with its design motivation: high-aspect-ratio ships are more sensitive to angular deviations than compact targets. When the two components are used together, the model achieves the best overall performance, showing that feature-level adaptation and angle-level supervision are complementary rather than redundant.

Compared with the Baseline, YOLO-CPCL produces more correct detections and reduces both missed detections and background false positives on HRSC2016. In contrast, the number of inter-class confusions changes only slightly. This pattern indicates that the proposed method mainly improves target preservation and background interference suppression. It does not rely on a broad reduction of confusion among visually similar ship categories. The feature-response visualizations are also consistent with this observation: the responses of YOLO-CPCL tend to be more concentrated around ship bodies, whereas the Baseline shows more dispersed activations over water texture, shoreline structures, and port facilities. Figure 6 shows three recurrent failure modes: false positives caused by aligned shoreline structures, confusion between visually similar ship types, and missed detections in densely berthed scenes.

The ShipRSImageNet results show category-dependent gains. Commander, Patrol, Auxiliary Ships, and Tugboat improve markedly, whereas several merchant and visually similar categories show limited gains or moderate degradation. This indicates that the benefit of YOLO-CPCL varies across ship categories.

The custom DOTA-v1.0 experiment provides supplementary evaluation on a different aerial-image source. Both models are trained with all 15 categories, while ship-class results are reported for comparison.

The three-seed evaluation shows that YOLO-CPCL consistently outperforms the adopted baseline and exhibits lower variability in Recall and the mAP metrics. Practical tests on the NVIDIA GeForce GTX 1650 further show that the reductions in parameters and GFLOPs are accompanied by higher latency and memory usage and lower throughput. Thus, YOLO-CPCL is compact in structural complexity, although the current implementation does not reduce practical runtime cost.

## 6. Conclusions

This study developed YOLO-CPCL for multi-class oriented ship detection in optical remote sensing imagery. SOSA-Fuse replaces all four C2f fusion units in the Neck with C2f-style adaptive-sampling blocks, while ARCAS-Loss replaces the baseline auxiliary angle term with a bounded aspect-ratio-dependent periodic angle term during training. The changes are restricted to feature fusion and auxiliary supervision; the Backbone, detection scales, and original oriented-box regression loss are retained.

On the four-class HRSC2016 task, YOLO-CPCL improves Precision, Recall, mAP@50, mAP@75, and mAP@50–95 by 3.54, 7.39, 5.48, 8.60, and 7.23 percentage points, respectively. The parameter count decreases from 3.08 M to 2.92 M and GFLOPs from 8.3 to 7.6. Evaluations on ShipRSImageNet and the ship-class results under the custom DOTA-v1.0 protocol also show positive aggregate changes, with the clearest gains generally appearing in recall and strict-IoU localization. Class-wise results, however, remain uneven, and class confusion is not consistently reduced.

Future work will focus on further reducing the runtime overhead of adaptive sampling on resource-constrained hardware and improving fine-grained recognition for rare and visually similar ship categories. Long-tailed category learning and standardized cross-dataset protocols are also needed before broader claims about fine-grained recognition or domain generalization can be made.

## Figures and Tables

**Table 1:** Data split and category statistics of the HRSC2016 four-class task.

Split	Images	Aircraft Carrier	Warship	Merchant Ship	Submarine	Total Objects
Train	436	63	607	218	32	920
Val	181	29	308	93	12	442
Test	444	64	637	229	60	990
Total	1061	156	1552	540	104	2352

**Table 2:** Datasets and experimental settings.

Dataset	Original Characteristics	Setting in This Paper	Split/Preprocessing Protocol	Role in This Paper
HRSC2016	A high-resolution optical ship dataset with oriented annotations	A four-class oriented ship detection task is constructed	Training, validation, and testing are conducted according to the official train/val/test protocol	Main experimental platform
ShipRSImageNet	A hierarchical fine-grained ship detection dataset	The Level-2 hierarchy is adopted; 24 classes are retained after removing DOCK	The official train/val/test split is used; empty-label images are removed; annotations are converted into YOLO-OBB format	Additional multi-class evaluation
DOTA-v1.0	A large-scale aerial-image dataset with 15 oriented-object categories	All 15 categories are retained for training; only ship-class metrics are reported	The original training set is repartitioned into 80/20 subsets; the original validation set is used as the test set; images are center-cropped or padded to 1024 × 1024; the network input is 640 × 640; annotations are converted to YOLO-OBB format	Controlled ship-class comparison under a custom protocol

**Table 3:** Performance comparison with lightweight YOLO series.

Model	Precision (%)	Recall (%)	mAP@50 (%)	mAP@75 (%)	mAP@50–95 (%)	Params (M)	GFLOPs
YOLOv8n-OBB	**79.31**	72.95	**79.93**	**71.34**	**61.75**	3.08	8.3
YOLOv11n-OBB	75.11	76.17	79.18	69.94	60.28	2.65	6.6
YOLOv12n-OBB	67.32	**77.91**	78.19	68.91	59.72	2.63	6.6
YOLO26n-OBB	60.34	64.16	66.14	59.93	51.24	**2.45**	**5.4**

Note: Bold values indicate the highest accuracy value or the lowest complexity value in each corresponding column.

**Table 4:** Sensitivity analysis of different combinations of α and $W_{\max}$ for the Baseline + ARCAS-Loss configuration on the HRSC2016 test set.

α	$W_{\max}$	Precision (%)	Recall (%)	mAP@50 (%)	mAP@75 (%)	mAP@50–95 (%)
0.50	1.5	77.61	76.24	79.62	73.93	61.88
0.50 ^†^	2.0	72.90	**79.15**	81.93	72.98	**63.45**
0.50	3.0	78.01	74.74	80.50	**74.52**	63.01
0.25	1.0	**81.90**	77.79	**82.18**	71.00	63.19
0.25	2.0	78.78	72.86	80.07	68.12	61.53
0.25	3.0	78.78	72.86	80.07	68.12	61.53
0.75	2.0	76.39	72.75	79.42	70.13	60.98
1.00	2.0	78.11	74.55	80.18	72.00	62.13

^†^ The setting selected for the final YOLO-CPCL model. Bold and underlined values in the metric columns indicate the best and second-best results, respectively.

**Table 5:** Comparison with GWD-based box regression and angle-supervision variants on the HRSC2016 test set (%).

Variant	Box Term	Angle Term	Precision (%)	Recall (%)	mAP@50 (%)	mAP@75 (%)	mAP@50–95 (%)
Baseline	ProbIoU + DFL	Baseline angle	79.31	72.95	79.93	71.34	61.75
GWD	GWD + DFL	Baseline angle	76.81	75.10	78.57	68.39	59.10
Cosine	ProbIoU + DFL	Cosine	81.90	77.79	82.18	71.00	63.19
ARCAS-Loss	ProbIoU + DFL	ARCAS	72.90	79.15	81.93	72.98	63.45

Note: GWD: Gaussian Wasserstein Distance; DFL: Distribution Focal Loss; Baseline angle: the original auxiliary angle-supervision term; Cosine: the unweighted periodic cosine angle-loss variant; ARCAS: the bounded aspect-ratio-coupled periodic cosine angle-loss formulation.

**Table 6:** Ablation experiments of the proposed modules on HRSC2016.

No.	Baseline	SOSA-Fuse	ARCAS-Loss	Precision (%)	Recall (%)	mAP@50 (%)	mAP@75 (%)	mAP@50–95 (%)	Params (M)	GFLOPs
0	✓	—	—	79.31	72.95	79.93	71.34	61.75	3.08	8.3
1	✓	✓	—	75.03	79.92	82.57	74.95	65.80	**2.92**	**7.6**
2	✓	—	✓	72.90	79.15	81.93	72.98	63.45	3.08	8.3
3	✓	✓	✓	**82.85**	**80.34**	**85.41**	**79.94**	**68.98**	**2.92**	**7.6**

Note: A check mark (✓) indicates that the corresponding module is included. Bold values indicate the best results.

**Table 7:** Component analysis of content-adaptive sampling in SOSA-Fuse on the HRSC2016 test set.

Variant	C2f-Style Topology	Adaptive Sampling	Precision (%)	Recall (%)	mAP@50 (%)	mAP@75 (%)	mAP@50–95 (%)
YOLOv8n-OBB	✓	–	79.31	72.95	79.93	71.34	61.75
SOSA-Fuse w/o adaptive sampling	✓	–	80.50	75.30	79.99	72.18	61.35
Full SOSA-Fuse	✓	✓	75.03	79.92	82.57	74.95	65.80

**Table 8:** Ablation study of the SOSA-Fuse replacement ratio under a nested Neck-location strategy on the HRSC2016 test set.

Variant	Replaced C2f Indices	Ratio	Precision (%)	Recall (%)	mAP@50 (%)	mAP@75 (%)	mAP@50–95 (%)
Baseline	None	0%	79.31	72.95	79.93	71.34	61.75
SOSA-25%	15	25%	80.27	72.85	79.79	72.58	63.03
SOSA-50%	12, 15	50%	77.39	75.38	81.41	72.87	63.43
SOSA-75%	12, 15, 21	75%	**83.47**	71.22	81.87	74.35	64.17
SOSA-100%	12, 15, 18, 21	100%	75.03	**79.92**	**82.57**	**74.95**	**65.80**

Note: Bold values indicate the best result in each metric column.

**Table 9:** Comparison of model complexity and inference efficiency on the HRSC2016 test set.

Model	Params (M)	GFLOPs	Total Latency (ms/Image)	FPS	Peak Allocated GPU Memory (MB)
YOLOv8n-OBB	3.08	8.3	16.53	60.51	225.29
YOLO-CPCL	2.92	7.6	20.79	48.09	257.56

**Table 10:** Performance of the baseline and YOLO-CPCL over three random seeds on the HRSC2016 test set.

Model	Seed	Precision (%)	Recall (%)	mAP@50 (%)	mAP@75 (%)	mAP@50–95 (%)
Baseline	0	79.31	72.95	79.93	71.34	61.75
Baseline	1	72.35	74.40	77.13	66.81	59.48
Baseline	2	70.73	75.97	76.17	67.81	59.01
Baseline	Mean ± SD	74.13±4.56	74.44±1.51	77.74±1.95	68.65±2.38	60.08±1.47
YOLO-CPCL	0	82.85	80.34	85.41	79.94	68.98
YOLO-CPCL	1	78.52	80.59	84.30	80.03	67.46
YOLO-CPCL	2	77.19	81.35	84.98	79.70	67.74
YOLO-CPCL	Mean ± SD	79.52±2.96	80.76±0.53	84.90±0.56	79.89±0.17	68.06±0.81

Note: All values are reported as percentages. SD denotes the sample standard deviation.

**Table 11:** Comparison of category-level mAP@50 (%) results for the four ship classes on HRSC2016.

Class	Baseline	Baseline+SOSA-Fuse	Baseline+ARCAS-Loss	YOLO-CPCL
Aircraft carrier	87.56	91.38	86.74	**95.02**
Warship	93.44	**94.76**	94.11	94.26
Merchant ship	58.04	60.74	**62.04**	61.84
Submarine	80.66	83.42	84.84	**90.51**

Note: Bold and underlined values indicate the best and second-best results in each row, respectively.

**Table 12:** Comparison of category-level mAP@75 (%) results for the four ship classes on HRSC2016.

Class	Baseline	Baseline+SOSA-Fuse	Baseline+ARCAS-Loss	YOLO-CPCL
Aircraft carrier	84.35	89.78	85.61	**95.02**
Warship	92.18	**93.84**	91.98	93.24
Merchant ship	53.38	57.18	**60.18**	60.14
Submarine	55.44	58.99	54.15	**71.37**

Note: Bold and underlined values indicate the best and second-best results in each row, respectively.

**Table 13:** Comparison of category-level mAP@50–95 (%) results for the four ship classes on HRSC2016.

Class	Baseline	Baseline+SOSA-Fuse	Baseline+ARCAS-Loss	YOLO-CPCL
Aircraft carrier	73.02	77.85	72.70	**82.56**
Warship	79.87	**82.14**	80.30	81.93
Merchant ship	45.94	50.16	**51.20**	51.02
Submarine	48.15	53.07	49.62	**60.41**

Note: Bold and underlined values indicate the best and second-best results in each row, respectively.

**Table 14:** Comparison between the proposed method and existing oriented object detection methods on the HRSC2016 four-class task.

Model	Stage	Backbone	mAP@50 (%)	Params (M)	GFLOPs
Rotated Faster R-CNN	Two-stage	R50	70.10	41.13	78.12
RoI Transformer	Two-stage	R50	78.10	55.05	79.16
R3Det	One-stage	R50	75.10	41.65	128.99
S^2^A-Net	One-stage	R50	81.20	38.55	76.76
Oriented R-CNN	Two-stage	R50	83.10	41.13	78.17
YOLO-CPCL	One-stage	YOLOv8n	**85.41**	**2.92**	**7.6**

Note: Bold values indicate the highest mAP@50 and the lowest parameter count and GFLOPs among the compared methods.

**Table 15:** Error-type statistics based on the confusion matrices of Baseline and YOLO-CPCL on the HRSC2016 test set.

Method	Correct Detections	Missed Detections	Background False Positives	Class Confusion
Baseline	803	135	238	52
YOLO-CPCL	831	106	222	53
Change	+28	−29	−16	+1

**Table 16:** Aspect-ratio-grouped target-recovery and localization results on the HRSC2016 test set.

AR Group	No. of Targets	Model	Recall@50 (%)	Recall@75 (%)	Matched Mean IoU
AR<2	4	YOLOv8n-OBB	25.00	0.00	0.7051
AR<2	4	YOLO-CPCL	25.00	25.00	0.7609
2≤AR<4	105	YOLOv8n-OBB	80.95	51.43	0.7794
2≤AR<4	105	YOLO-CPCL	80.00	61.90	0.8099
AR≥4	881	YOLOv8n-OBB	96.03	81.61	0.8358
AR≥4	881	YOLO-CPCL	96.14	86.15	0.8526

**Table 17:** Ablation experiments of the proposed modules on ShipRSImageNet.

No.	Baseline	SOSA-Fuse	ARCAS-Loss	Precision (%)	Recall (%)	mAP@50 (%)	mAP@75 (%)	mAP@50–95 (%)
0	✓	—	—	52.59	49.10	50.77	45.28	40.29
1	✓	✓	—	58.90	52.14	54.77	49.29	44.00
2	✓	—	✓	51.24	51.77	50.11	44.77	39.43
3	✓	✓	✓	**59.01**	**54.36**	**55.46**	**49.39**	**44.35**

Note: A check mark (✓) indicates that the corresponding module is included. Bold values indicate the best results.

**Table 18:** Comparison of category-level mAP@50 (%) results on ShipRSImageNet.

Class	Baseline	Baseline+SOSA-Fuse	Baseline+ARCAS-Loss	YOLO-CPCL
Aircraft carrier	94.42	96.25	92.57	**97.33**
Cruiser	80.72	**88.93**	80.03	88.01
Destroyer	87.66	**91.27**	87.80	90.24
Frigate	77.55	**83.76**	80.80	83.60
Patrol	7.52	6.38	8.02	**24.35**
Landing	84.55	85.81	77.94	**87.12**
Commander	23.86	58.37	30.67	**60.49**
Auxiliary Ships	53.28	62.24	54.36	**64.61**
Submarine	92.13	91.57	90.30	**92.92**
Other Warship	47.13	**48.67**	45.20	47.92
Other Merchant	20.06	**21.21**	20.83	18.03
Container Ship	47.27	41.05	43.39	**51.61**
RoRo	95.21	**97.84**	94.47	96.72
Cargo	**70.41**	69.92	62.13	63.04
Barge	31.37	**37.43**	28.60	32.52
Tugboat	31.07	36.18	36.85	**42.22**
Ferry	19.58	20.94	**21.31**	20.63
Yacht	39.41	**45.47**	29.77	41.50
Sailboat	18.55	**32.41**	17.60	32.00
Fishing Vessel	12.15	**14.16**	13.61	10.95
Oil Tanker	54.85	**62.84**	52.81	52.44
Hovercraft	79.26	77.70	**79.33**	77.31
Motorboat	24.60	16.93	28.51	**30.91**
Other Ship	26.00	**27.06**	25.70	24.66

Note: Bold and underlined values indicate the best and second-best results in each row, respectively.

**Table 19:** Comparison of category-level mAP@50–95 (%) results on ShipRSImageNet.

Class	Baseline	Baseline+SOSA-Fuse	Baseline+ARCAS-Loss	YOLO-CPCL
Aircraft carrier	87.11	88.12	83.67	**90.56**
Cruiser	67.45	**75.83**	68.52	75.31
Destroyer	75.75	**79.79**	75.39	78.40
Frigate	65.33	**71.44**	66.97	69.89
Patrol	6.28	5.30	6.78	**20.86**
Landing	69.91	71.36	63.21	**72.08**
Commander	21.74	54.12	28.23	**56.86**
Auxiliary Ships	46.95	55.79	48.34	**57.77**
Submarine	52.63	**53.77**	49.41	53.43
Other Warship	37.42	38.62	36.04	**38.83**
Other Merchant	15.10	**17.02**	15.63	14.01
Container Ship	35.75	33.22	34.36	**40.74**
RoRo	84.88	**86.78**	80.84	84.98
Cargo	57.23	**58.47**	50.58	53.36
Barge	22.66	**26.40**	22.67	23.53
Tugboat	24.38	27.79	28.72	**32.80**
Ferry	14.56	15.62	**15.67**	13.73
Yacht	30.13	**34.09**	20.39	31.95
Sailboat	6.68	11.83	5.30	**12.58**
Fishing Vessel	7.68	**8.01**	7.88	6.30
Oil Tanker	46.56	**51.88**	43.89	43.43
Hovercraft	**65.31**	64.35	65.09	64.41
Motorboat	5.46	4.82	8.40	**9.10**
Other Ship	20.02	**21.61**	20.25	19.48

Note: Bold and underlined values indicate the best and second-best results in each row, respectively.

**Table 20:** Ship-class results of YOLOv8n-OBB and YOLO-CPCL under the custom DOTA-v1.0 protocol (%).

Method	Precision (%)	Recall (%)	mAP@50 (%)	mAP@75 (%)	mAP@50–95 (%)
Baseline	91.53	73.26	82.85	47.41	47.77
YOLO-CPCL	**91.93**	**75.81**	**83.85**	**50.09**	**49.84**

Note: Bold values indicate the better result in each metric column.

## Data Availability

The datasets analyzed in this study are publicly available from the original sources cited in the manuscript. The processed annotations, class-mapping rules, and evaluation protocols are available from the corresponding author upon reasonable request.

## Abbreviations

The following abbreviations are used in this manuscript:

Abbreviation	Full Name
YOLO	You Only Look Once
OBB	Oriented Bounding Box
SOSA-Fuse	Ship-Oriented Slender Adaptive Fusion
ARCAS-Loss	Aspect-Ratio-Coupled Angle Supervision Loss
C2f	CSP Bottleneck Module with Two Convolutions
DCN	Deformable Convolutional Network
FPN	Feature Pyramid Network
PANet	Path Aggregation Network
BiFPN	Bidirectional Feature Pyramid Network
DETR	Detection Transformer
RT-DETR	Real-Time Detection Transformer
CSL	Circular Smooth Label
GWD	Gaussian Wasserstein Distance
ProbIoU	Probabilistic Intersection over Union
KFIoU	Kalman Filter Intersection over Union
DFL	Distribution Focal Loss
BN	Batch Normalization
IoU	Intersection over Union
AP	Average Precision
mAP	Mean Average Precision
FPS	Frames per Second
GFLOPs	Giga Floating-Point Operations
GT	Ground Truth
SAR	Synthetic Aperture Radar
UAV	Unmanned Aerial Vehicle
TF32	TensorFloat-32
